# A Linear Ablating System in the Left and Right Atrium: Feasibility, Catheter Performance and Clinical Results

**DOI:** 10.1016/s0972-6292(16)30500-9

**Published:** 2012-05-20

**Authors:** Aruna Arujuna, Cliona Murphy, Azmat Hayat, Hollie Seffens, Jaswinder S Gill

**Affiliations:** Cardiothoracic Centre, Guy's and St Thomas' Hospitals, London, UK

**Keywords:** Atrial fibrillation, linear ablation, catheter performance, clinical outcome

## Abstract

**Introduction:**

We describe the use of a ablating system to compartmentalise and regionally isolate the atria in paroxysmal and persistent atrial fibrillation (AF).

**Methods:**

40 patients were studied, 25 paroxysmal AF and 14 persistent AF. One patient enrolled was later found to be in left atrial flutter and was excluded. The Cardima Revelation® TX catheter system with Intellitemp® Radiofrequency (RF) energy control device and a Medtronic Atakar® RF generator were used to place wide area circumferential ablations to achieve conduction block into the left and right sided pulmonary veins. Roof lines and mitral isthmus lines were also performed. In patients with persistent AF and in repeat procedures, right atrial compartmentalisation was performed with an anterior superior vena cava (SVC) to inferior vena cava (IVC) line and a septal SVC to IVC line.

**Results:**

At 6 months, 18 of the 39 patients were asymptomatic, 10 had improved symptoms and 22 were in sinus rhythm. In the paroxysmal group, 11 were asymptomatic, 7 had improved symptoms and 16 (64%) were in sinus rhythm. In the persistent group, 7 were asymptomatic, 3 had improved symptoms and 6 (43%) were in sinus rhythm. The total group AF burden was 37.8 ± 5.4 hrs pre-procedure and 23.1 ± 5.1 hrs at 6 months post procedure. Mean temperature, impedance and power recorded at each pole demonstrated effective power delivery at all poles. No catheter charring was observed, complication rates were comparable to standard AF ablation technique.

**Conclusion:**

Linear ablation in the left and right atria to mimic Cox's Maze is feasible and safe using this ablating system.

## Introduction

Paroxysmal atrial fibrillation (AF) is frequently initiated by triggers in the pulmonary veins. Pulmonary vein isolation (PVI) can eliminate the AF triggers from entering the left atrium and prevent occurrence of AF [1]. Linear ablation theoretically creates "lines of block" which disperse the refractory periods hence reducing the capability of atrium to sustain AF [2]. Surgically deployed intra-operative linear atrial lines, as in Cox's Maze operation [3] are highly effective in reducing the occurrence of AF even in patients in very diseased atria [4,5]. This has led to the presumption that the same success can be achieved with catheter based techniques, however these attempts have met with variable success [6]. Procedure times are long and it can be difficult to ascertain whether an ablation line is complete. Even if a line appears complete at the time of the procedure, gaps can appear in the subsequent healing process leading to recurrent arrhythmia. Incomplete linear lesions can be pro-arrhythmic and predispose to new arrhythmias, in particular left atrial flutter [7]. Investigators reported improved ablation success rates by the addition of linear lines to the PVI procedure, in particular the mitral isthmus line [8] and the left atrial roof line [9]. A recent evaluation of the efficacy of LA linear ablation using a hexapolar linear multi-electrode mapping/ablation catheter has described safety and acute outcomes [10]. In this study, we propose to assess safety and 6 month clinical outcome following PVI and linear ablation using a multi-electrode ablation catheter to create continuous linear lines in the left and right atria. This study is a preliminary report of the use of the Cardima Revelation T-flex ablating system® for this purpose. The approach is similar to the Cox Surgical Maze, but is less invasive, therefore carrying a lower risk of complication and a shorter recovery time.

## Catheter ablation system - description

### i) Linear ablation catheter (Revelation T-flex ®)

The Revelation T-flex ® linear ablation catheter is a single use deflectable multi-electrode electrophysiology catheter designed to record intracardiac potentials and deliver radiofrequency energy for cardiac tissue ablation. The catheter has a linear configuration and is deflectable in one plane (Figure 1a), measuring 150cm in length with electrode spacing of 2mm. The shaft has 16 signal recording electrodes and 16 ablation coils, each 3mm in length, with dual thermocouples at each electrode. The flexible coiled electrodes are integrated into the catheter and create deep continuous curvilinear transmural lesions. The thin lesions result in less surrounding myocardial tissue damage. Thermocouples are integrated between electrodes to sense tissue temperature and sensing occurs through the coils. The catheter has a single plane of deflection and RF energy can be delivered through up to 8 electrodes simultaneously. The catheter has an atraumatic platinum tip coil for added safety. The catheter dimensions are: outer diameter 5.5 French, working length 130cm, curve reach 4cm, electrode length 3mm. In comparison to standard "drag and burn" ablation technologies, simultaneous ablation through multiple electrodes of a linear ablation catheter may shorten procedure time, in theory creating lesions that have penetrating depth without gaps along the linear trajectory.

### ii) Deflectable guiding catheter/sheath (Naviport®)

The Naviport torqueable support sheath can be used with the Revelation ablation catheter to provide additional support deflectability and maneuverability. It has a radiopaque shaft with a soft atraumatic tip. (Figure 1b).

### iii) Energy Management Device (Intellitemp®)

The Intellitemp® Energy Management Device (EMD) is used in conjunction with the Revelation ablation catheter to control the delivery of radio-frequency (RF) energy during ablation. RF energy can be applied at individual electrodes or in a combination of up to 8 electrodes simultaneously (the proximal or distal set of the 16 electrodes or any combination of eight electrodes). Thermocouples provide direct temperature feedback from the tissue to the EMD which modulates the RF energy to each individual electrode based on the recorded temperature in order to create optimal lesion formation. The feedback system incorporates impedance in addition to temperature and impedance out of set limits will result in that pole being switched off during energy application. 

This study was performed to assess several outcomes. Firstly to study the feasibility of creating linear ablation lines in the left and right atria using the Revelation T-flex catheter ablating system and to perform an evaluation of the catheter performance, assessing RF energy delivery and temperatures achieved. The second aim was to assess the practical use of the system in routine clinical workflow by evaluation of procedural and screening times.

Next, outcome evaluation of both acute ablation success with this system and clinical follow-up data at 3 and 6 months post catheter ablation of symptoms, rate of recurrence of AF and AF burden. The final objective was to document the safety of this ablating catheter system.

## Methods

### Study patients

This prospective study was commenced following formal approval by the local hospital ethics committee. 40 consecutive patients with paroxysmal or persistent AF were enrolled for the study after informed written consent had been obtained. All patients enrolled into the study had documented symptomatic paroxysmal or persistent atrial fibrillation (AF) whilst taking one or more anti-arrhythmic medications. AF was defined as persistent if present for > 7 days or a DC cardioversion attempt had failed.

These patients in AF had associated recognizable symptoms such as shortness of breath, palpitations, lightheadedness or fatigue. 'Refractory' AF was defined as a failed rhythm control after trial of two or more anti-arrhythmic drugs (AAD) or Amiodarone alone. AAD failure was defined as the occurrence of AF after taking AADs for at least one month. In all patients the AAD regimen had to be stable without changes in the regimen/dosage in the previous month. The following exclusion criteria were applied - asymptomatic AF patients, patients unable to take formal anticoagulation therapy pre/post procedure or the presence of a left atrial clot seen on pre-procedure trans-oesophageal echocardiogram.

### Patient symptoms and AF burden

In each patient symptoms were documented and AF burden measured by 72 hour Holter monitoring (Getemed GE) at three clinic visits; before the procedure, three months and six months post procedure. Holter recordings were analysed using a Cardioday software system (GE version 11.0 for windows) with visual verification.

### Ablation procedure protocol

Two trans-septal punctures were performed to access the left atrium and angiograms of the left and right sided pulmonary veins were taken with contrast injection. Through the trans-septal sheaths an Optima Inquiry® catheter (St Jude Medical) and the T-flex ablation catheter, were deployed in the left atrium (Figure 2a,2b,2c,2d). Left atrial geometry was created using the NavX system (Ensite/St Jude) with a 6 French screw in reference catheter positioned in the right atrium as a geometry reference. The activated clotting time was maintained between 250 and 300 sec with intravenous heparin throughout the procedure.

The Revelation T-flex ablation catheter with the Intellitemp energy management device and Medtronic Atakar radiofrequency generator were used to deliver radiofrequency ablation. The settings for energy delivery were temperature set at 50ºC for 60-90 seconds with an impedance range of 190 to 247 ohms. Wide area circumferential ablations were performed to achieve conduction block into the left and right sided pulmonary veins, confirmed by the absence of significant signals within each vein post ablation. The Optima Inquiry® catheter (St Jude Medical) was used pre and post ablation to confirm successful pulmonary vein isolation. Coronary sinus pacing was also undertaken where necessary for confirmation of block. Independent pulmonary vein potentials in the absence of atrial capture confirmed pulmonary vein isolation. Linear ablation lines were next drawn along the roof between the left and right superior veins and from the left lower pulmonary vein to the mitral valve annulus. Confirmation of block across the two ablation lines was not performed. Additionally, in patients with persistent atrial fibrillation or redo procedures, right atrial compartmentalisation with an anterior SVC-IVC line and septal SVC-IVC line was performed. Confirmation of block across the right atrial lines was not attempted. However, in this department we have previously performed right atrial linear ablation for compartmentalisation, confirming conduction block after ablation by pacing on either side of the line and following the conduction wavefront with an Ensite array® (St Jude) deployed in the right atrium. The ablation catheter performance was assessed in the first 29 patients studied. Catheter temperature (ºC), power (Watts) and impedance (Ohms) were recorded at each pole for each application, with mean values then calculated per pole and per patient.

### Follow-up

Two follow-up visits were performed, at three and six months post procedure. Patients remained on their previous anti-arrhythmic medication for at least 3 months after the ablation procedure and those in stable sinus rhythm after 6 months had anti-arrhythmic medication discontinued or reduced as appropriate. Anticoagulation post procedure was continued with warfarin.

### Data Analysis

Categorical variables are expressed as frequencies and percentages. Continuous data are presented as mean value ± standard deviation (SD) or median. Continuous variables were analysed with the student's T-test and a value of less than 0.05 was considered statistically significant.

## Results

The study was carried out at St Thomas Hospital between Jan 2007 and Jan 2009 and all 39 patients completed 6 months of follow up.

### Patient characteristics

39 patients, 27 male were studied and underwent an ablation procedure. The mean patient age was 60.8 years (range 39 - 77 yrs), mean duration of AF 7.5±0.9 years and average LA size 4.2±1.0 cm. At enrolment 25 patients had paroxysmal AF and 14 had persistent AF. 12 patients had undergone a previous pulmonary vein isolation procedure. One patient enrolled was found subsequently at electrophysiological study to have left atrial flutter and was not included further in the study. Patient characteristics including number of previous AADs failed are shown in Table 1.

### Acute ablation success

All 4 major pulmonary veins were isolated in all but one patient in whom it was not possible to isolate the right lower pulmonary vein.

### Patient symptoms

In the total group 18 of the 39 patients were asymptomatic at 6 months, 10 had reduced symptoms and 11 remained symptomatic. The corresponding figures for paroxysmal and persistent AF are shown in Table 2. This demonstrates that more than 70% of patients in both paroxysmal and persistent AF reported an improvement in their symptoms. Interestingly, 50% of the persistent AF group were asymptomatic compared to 44% in the paroxysmal AF group.

### AF burden, anti-arrhythmics and sinus rhythm at follow up

The number of patients in sinus rhythm at 3 and 6 months in the paroxysmal and persistent groups and the AF burden from the Holter data are also shown in Table 2. At 6 months, 18 of the 39 patients were asymptomatic, 10 had improved symptoms and 22 were in sinus rhythm. In the paroxysmal group, 11 were asymptomatic, 7 had improved symptoms and 16 (64%) were in sinus rhythm. In the persistent group, 7 were asymptomatic, 3 had improved symptoms and 6 (43%) were in sinus rhythm. At 6 months, all 16 PAF patients who were in sinus rhythm came off anti-arrhythmics whilst those with recurrences (9 patients) were maintained on their baseline medication: 7 patients on Beta-blockers, 5 on flecainide and 2 on calcium channel blockers (4 with one anti-arrhythmic and 5 on two anti-arrhythmics). 3 patients came off amiodarone. In the persistent AF group, 9 patients came off amiodarone whilst the others were maintained on their baseline anti-arrhythmics: 12 on beta-blockers, 3 on amiodarone, 2 on calcium channel blockers and 2 on digoxin (4 on a single anti-arrhythmic, 3 with two anti-arrhythmics and 3 on three anti-arrhythmics). There was a significant reduction in AF burden in the persistent AF group at 6 months (P<0.01) with 43% of this group holding sinus rhythm throughout the 3 day holter. Two patients had gap related atrial flutter on their 3 month Holter monitor. All patients with recurrences were listed for a re-do procedure.

### Procedural times and catheter performance data

The mean procedural time was 217 ± 12 minutes with a mean fluoroscopy time of 37 ± 2 minutes. The average number of RF applications per patient was 52 (range 12 to 90). Dichotomising procedural times into paroxysmal and persistent AF groups, the values are 206 ± 15 minutes and 248 ± 10 minutes respectively.

**Figure 3:**
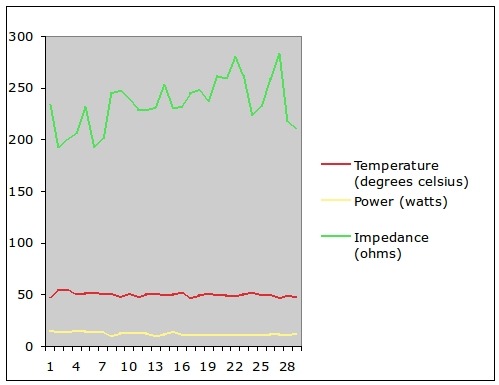
blation catheter temperature, power and impedance - mean values in the first twenty nine study patients. Means ±SEM - Power=11.9±0.3W, Temperature=49.9±0.4 ºC, Impedance=227.7±8.5 ohms

The mean catheter temperature, impedance and watts achieved in the first 29 study patients are shown in Figure 3. The mean temperature achieved was 49.9±0.4 ºC, mean power delivered was 11.9±0.3 W and mean impedance encountered 227.7±8.5 ohms. A tabulated recording of this similar means achieved at each of the 16 catheter poles for the same study patients are presented in Table 3.

### Complications and safety

No direct complication related to the catheter was observed. Two patients developed acute back burns, attributed to incorrectly sized back pads. After this problem was identified correct back pads were used in all subsequent cases and no further burns were observed. 2 patients had cardiac tamponade requiring pericardiocentesis. 2 patients developed gap related left atrial flutter requiring catheter ablation. A third patient has developed left atrial flutter after completion of the study period. No patient suffered a transient ischemic attack or stroke. Catheter charring was not observed during any of the procedures.

## Discussion

### AF burden/Clinical outcome/Symptoms

In this study the AF burden was found to be significantly reduced for the total study and persistent AF groups following linear ablation. Although statistically non significant there was also a reduction in AF burden at 6 months in the paroxysmal group. Out of 39 patients, only three patients had a higher AF burden post than pre procedure. Of particular note is that almost half the persistent AF patients, all of whom were in AF pre-procedure, were holding sinus rhythm at 6 months after a single procedure. Interestingly in our study cohort, persistent patients who failed the ablation procedure universally returned to AF and none converted from persistent to paroxysmal AF.

The proportion of patients in the paroxysmal group demonstrating improved symptoms was greater than in the persistent group. However the reduction in AF burden on Holter was not as high as expected given this improvement in symptoms. Explanations for symptomatic improvement despite continuing AF could include ganglionic plexus modification or placebo effect.

### Catheter performance

The presented data suggests that the Cardima Revelation linear ablation catheter system can be used to achieve acute pulmonary vein isolation. Conduction block into the pulmonary veins is strongly suggestive of effective lesion application and this end point which was convincingly demonstrated in all but one patient. The energy delivered at each pole of the catheter during the 60 to 90 second applications appears to be sufficient to achieve effective tissue destruction at the respective application site. Indeed split potentials and diminished electrograms demonstrable in some but not all cases suggesting an adequate lesion depth has been achieved at each pole. The Intellitemp system was documented to deliver sufficient energy at up to 8 catheter poles at a time. The Intellitemp automatically notes if a pole goes out of range for temperature, power or impedance and appropriately responds for each ablation point, ie if impedance becomes high it will switch off power to that pole. This is indicated on the output of the device allowing for further ablation to be performed again if necessary. The power delivered at the poles of the ablation catheter were generally much lower than single pole ablation. Two reasons behind this include firstly temperature was limited to 50 degrees to avoid excessive overheating and potential charring. Secondly, the generator was limited to 100W such that on multiple poles limited power was delivered although in general target temperatures were not always achieved. This suggests that a generator capable of delivering more RF energy than the 100W delivered through the Medtronic Atakar may be more effective. For this study, given this being the first clinical setting in utilising the catheter and for safety reasons, a more conservative lower power delivery was employed. Future evaluation of cautious up-titration of power delivery would be needed. The target temperature and power settings in this study was the same for each pole and similar settings were used for PV isolation and all lines (including roof lines). Catheter manipulation can be difficult in some cases due to its longer length and may explain why the procedural times observed were not shorter than conventional burn and drag techniques in our laboratory. Manipulation of this catheter might be improved with operator experience following the learning curve and with the use of a steerable sheath (Cardima Naviport ®).

### Acute results

In this study the pulmonary veins were isolated in all but one case. The right lower pulmonary vein was the most difficult to isolate. This was on some occasion undertaken using the tip of the catheter (poles 1 and 2) to deliver directed local lesions. In some cases the catheter structure became distorted, but removal of catheter with manual reshaping was feasible. Linear application in the right atrium was easier to perform than in the left atrium and placement of the right sided lesions took less than 20 minutes. In terms of procedural time, this linear ablation catheter technique was comparable to previous data using standard ablation catheters/ablation techniques. The procedural time in the persistent patients with the addition of a RA MAZE was not longer than in those with PAF. This may be because in many of the persistent patients there was not much signal in the pulmonary veins at the start, with most of the procedure then directed towards bi-atrial compartmentalisation. The addition of a RA MAZE only added on average 20 mins to the total procedure time.

### Safety

The catheter proved to be safe in this study as evident by no embolic complications. The lack of charring of the catheter may partly explain this observation. This study demonstrates that the catheter can be used safely in the left atrium. Using the correct back pads is necessary with this catheter given the significant energy delivered through them. Smaller back pads were inadvertently placed for procedures on 2 patients resulting in superficial back burns. Subsequently in every case two back plates were used and no further burns occurred. The rates of tamponade and of LA flutter were similar to those described with conventional techniques (2-4%) [11,12]. Whilst no odynophagia and formal oesophageal injury was reported, an evaluation for potential injury was not performed. The current standard during RF ablation for AF utilizes irrigated tip catheters. The absence of irrigation in this catheter did not result in any subsequent clinical diagnosis of thrombo-embolic events although no formal evaluation of cerebral blood flow was performed.

### Study Limitations

The main limitation of this study was that not all ablation lines were checked and block confirmed by pacing techniques. In determining post procedure AF burden, study patients were only monitored for 72 hours. 24 to 72 hours of monitoring of Holter monitoring has been recommended by the HRS/ACC/ESC societies for monitoring AF burden in the follow up of subjects in AF ablation trials, as documented in their 2007 consensus statement on AF catheter ablation [13]. However it is quite possible that AF occurs outside this 'snapshot' time period and an implanted loop recorder would be useful for accurate follow up of AF burden for a more clinically relevant longer and continuous time period. Also longer term follow-up would be useful to evaluate post-ablation atrial tachycardia occurrence.

## Conclusion

In our initial experience, use of this linear ablating system within the left and right atrium to mimic Cox's MAZE procedure is feasible and safe. Further developments of this catheter and technique may reduce procedure times and increase therapeutic efficacy. A larger randomized study should be performed to evaluate the efficacy of this linear ablating system in assessing clinical outcomes.

## Figures and Tables

**Figure 1a:**
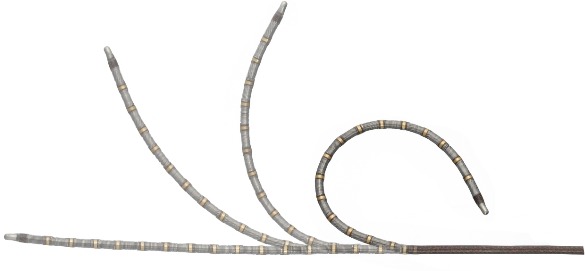
Image of the Cardima Revelation T-flex Linear Ablation Catheter depicting its curve reach sheath

**Figure 1b:**
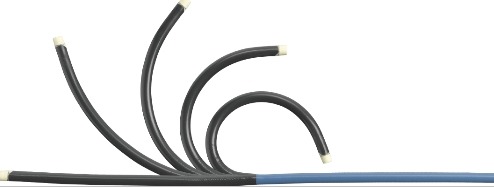
Image of the Cardima Naviport steerable sheath

**Figure 2a:**
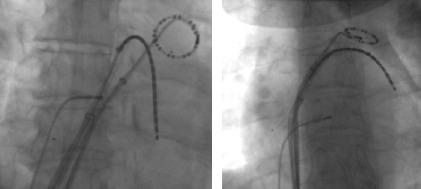
Ablation catheter position during WACA of the left pulmonary veins (LAO projection)

**Figure 2b:**
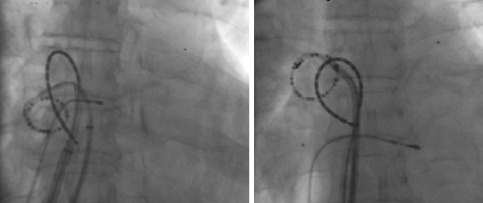
Ablation catheter position during WACA of the right pulmonary veins (AP projection)

**Figure 2c:**
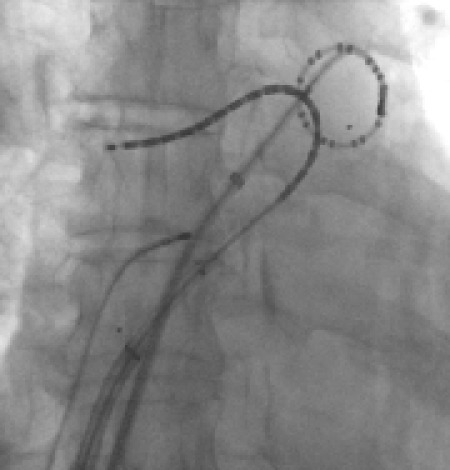
Ablation catheter placement during Left Atrial Roofline

**Figure 2d:**
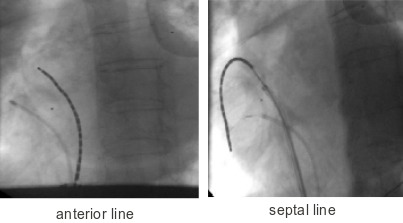
Ablation catheter position during right atrial linear ablation

**Table 1:**
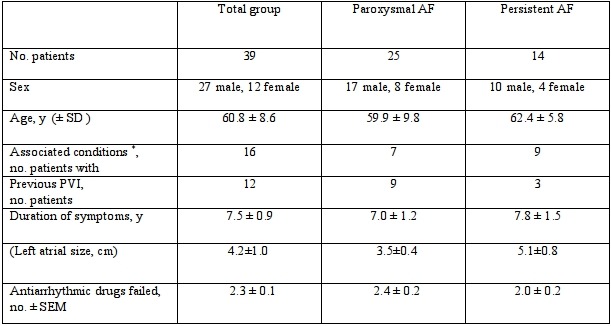
Patient characteristics

* Hypertension, diabetes mellitus, mitral valvular heart disease, heart failure

**Table 2:**
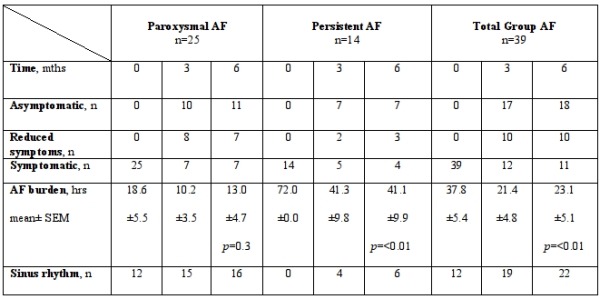
Patient symptoms and mean AF burden at 0, 3 and 6 months

N= number of patients. AF burden = hours in AF during 72 hour Holter. Sinus rhythm = sinus rhythm throughout 72 hour Holter. Students paired t-test was used to determine statistical significance.

**Table 3:**
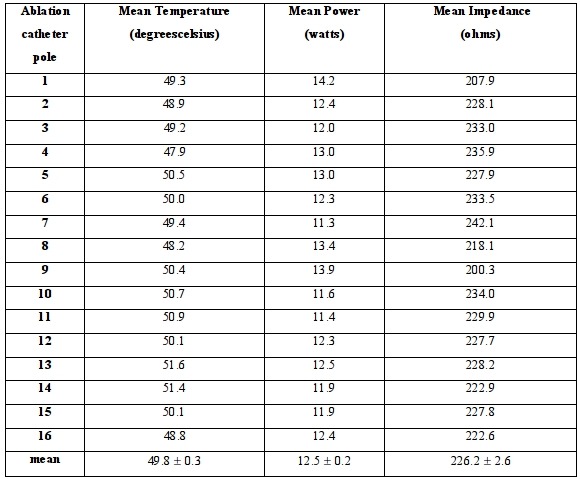
Ablation catheter temperature, power and impedance per each of the 16 poles in the first 29 study patients

Data = mean ± SEM
